# Cannabinoids: A New Group of Agonists of PPARs

**DOI:** 10.1155/2007/23513

**Published:** 2007-11-15

**Authors:** Yan Sun, Andy Bennett

**Affiliations:** School of Biomedical Sciences, University of Nottingham Medical School, Nottingham NG7 2UH, UK

## Abstract

Cannabinoids have been used medicinally and recreationally for thousands of years and their effects were proposed to occur mainly via activation of the G-protein-coupled receptor $\text{C}{\text{B}}_{1}/\text{C}{\text{B}}_{2}$ (cannabinoid receptor 1/2). Discovery of potent synthetic analogs of the natural cannabinoids as clinically useful drugs is the sustained aim of cannabinoid research. This demands that these new compounds be free of the psychotropic effects that connected with the recreational use of cannabinoids. In preclinical studies cannabinoids displayed many of the characteristics of nonsteroidal anti-inflammatory drugs (NSAIDs) and it seems to be free of unwanted side effects. An increasing number of therapeutic actions of cannabinoid are being reported that do not appear to be mediated by either $\text{C}{\text{B}}_{1}$ or $\text{C}{\text{B}}_{2}$, and recently nuclear receptor superfamily PPARs (peroxisome-proliferator-activated receptors) have been suggested as the target of certain cannabinoids. This review summarizes the evidence for cannabinoid activation on PPARs and possible associated remedial potentials.

## 1. INTRODUCTION

The term cannabinoid describes a group of compounds, which can potentially bind to the two recognised cannabinoid receptors (CB_1_/CB_2_). Many of these compounds are structurally related to $\Delta^{9}$-tetrahydrocannabinol (THC) (Mechoulam and Gaoni [1]), the major psychoactive component concentrated in the flowering head of the female plant of *Cannabis sativa* (marijuana), which has been used medicinally and recreationally for thousands of years. THC exhibits diverse pharmacological activities in vitro and in vivo. These responses include alterations in cognition and memory, euphoria, immobility, analgesia, hypothermia, and sedation (Howlett [2]). Although widely recognised as a drug of abuse, it is now apparent that THC, like heroin, mimics the functions of the endogenous cannabinoids, which have now been identified and appear to have roles in signalling in many different tissues including the central nervous system (CNS), immune system, and cardiovascular system (Mechoulam et al. [3]). The most prominent physiological effect of cannabis and other cannabinoids is the short-term ability to induce a state of euphoric intoxication in users. This intoxication is accompanied by slight impairment of psychomotor and cognitive function, a significant increase in heart rate and a decrease in blood pressure. In addition there is evidence that cannabinoids can alleviate spasticity, lower the heightened intraocular pressure associated with glaucoma, control the vomiting and nausea associated with cancer chemotherapy, and reduce pain. The long-term effects of cannabinoid exposure include alternations in the levels of male and female sexual hormones, possible teratogenic effects, immunosuppression, and possible physical dependence on the compounds (Cook et al. [4]).

**Figure 1:**
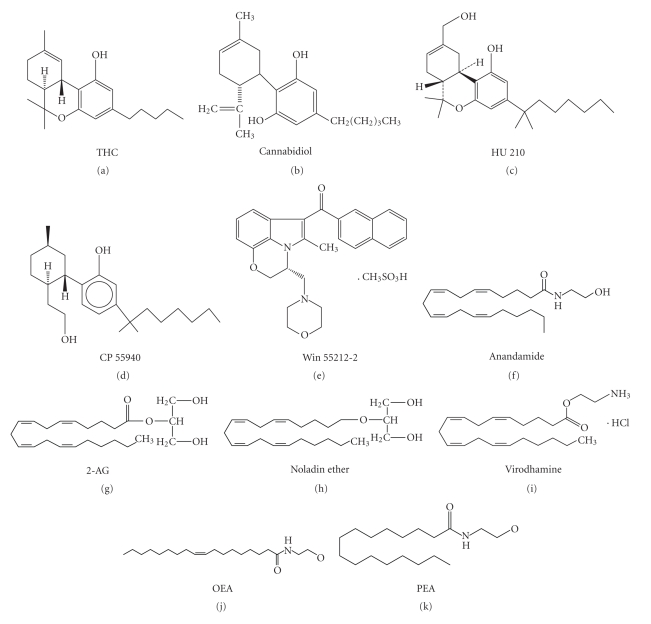
Chemical structures of cannabinoids. THC and cannabidiol are phytocannabinoids; HU 210, CP55940, and Win 55212-2 belong to the synthetic cannabinoid group; anandamide, 2-AG, noladin ether, virodhamine, OEA, and PEA are classed as endocannabinoids.

Based on their origins, cannabinoid receptor agonists can be classified into three groups. The first of these groups comprises the phytocannabinoids and includes THC and over 60 other cannabinoid compounds contained in *Cannabis sativa*. Cannabidiol (CBD) also belongs to this group and may modulate the response to THC by decreasing anxiety and antagonizing other THC-effects (Nadulski et al. [5]). The second group includes the synthetic cannabinoids that act on CB receptors. These are a diverse set of compounds which include dibenzopyran derivatives, such as HU210; some consists of bicyclic and tricyclic analogues of THC, which lack the central pyran ring common to the classical cannabinoid, such as CP55940; some consist of aminoalkylindoles, which are structurally very different from THC, such as Win 55212-2 (Pertwee [6]; Martin et al. [7]). The third cannabinoid group consists of arachidonic acid derivatives and contains all the currently recognised endogenous cannabinoids which are naturally occurring in vivo. Anandamide (arachidonylethanolamide) was the first identified endocannabinoid, which is able to reproduce the most typical behavioural effects of THC in rodents and was discovered in porcine brain in 1992 (Devane et al. [8]). Following the discovery of anandamide, 2-arachidonoylglycerol (2-AG), noladin ether and virodhamine (Lambert and Fowler [9]) have been found in the CNS. These compounds have cannabinoid receptor binding activity, but their exact physiological roles are unknown. Endocannabinoids have a short duration of action since they are rapidly metabolised by the intracellular enzymes such as fatty acid amide hydrolase (FAAH), monoglyceride lipase (MGL), or N-palmitoylethanolamine-selective acid amidase (NPAA). Some endogenous compounds such as palmitoylethanolamide (PEA) and oleoylethanolamide (OEA), which are structural analogues of anandamide, are also metabolised by these enzymes (Jonsson et al. [10]). Although they may not directly activate cannabinoid receptors, they have cannabimimetic effects and are regarded as cannabinoids by many researchers. There are also other standard criteria for the classification of cannabinoids, for example, based on their chemical structures, cannabinoids can be divided into classical cannabinoids, nonclassical cannabinoids, and endocannabinods (Figure 1).

Although many of the physiological responses to cannabinoids, such as analgesia, attenuation of nausea, and appetite stimulation are generally thought to be due to action at the CB_1_/CB_2_, studies in CB_1_, CB_2_, or CB_1_/CB_2_ double knockout mice have revealed non-CB_1_/CB_2_ receptor-mediated responses to cannabinoids, both in the CNS and periphery (Howlett et al. [11]). For example, anandamide and Win 55212-2, but not THC, stimulated [^35^S]GTPγS binding in the brains of CB_1_ knockout mice. This effect was not reversed by administration of CB_1_ and CB_2_ antagonists, SR 141716A or SR 144528, respectively, strongly suggesting the presence of novel non-CB_1_, non-CB_2_ cannabinoids receptors in the brain (Wiley and Martin [12]). Since the diversified structure of cannabinoids, their potential binding targets show divergence as well. For example, anandamide can bind vanilloid receptor (VR1) to produce membrane currents or increase intracellular calcium (Smart and Jerman [13]). PEA has been suggested to bind an SR144528-sensitive, non-CB_2_ receptor (“CB_2_-like” receptor) (Calignano et al. [14]). Evidence has also arisen for the existence of a brain G-protein-coupled receptor that can specifically bind Win55212-2 but not other CB_1_/CB_2_ agonists (Di Marzo et al. [15]; Breivogel et al. [16]). CBD has also been demonstrated as a modest affinity agonist of human 5-HT1α (5-hydroxytryptamine) receptor (Russo et al. [17]).

In addition to G-protein-coupled receptors (GPCR) and other plasma membrane receptors, another potential candidate for CB_1_/CB_2_-independent effects of cannabinoids is the PPAR (peroxisome-proliferator-activated receptor) family of nuclear receptor transcription factors. The three subtypes of PPARs (PPARα, PPARβ, and PPARγ) play important roles in regulation of lipid metabolism, hepatic peroxisomal enzyme expression, insulin sensitivity, glucose homeostasis, and inflammation.

The natural ligands for PPARs include fatty acids and eicosanoid derivatives (Table 1). In general, PPARγ apparently prefers polyunsaturated fatty acids such as linoleic acid, arachidonic acid, and eicosapentaenoic acid. Although most natural occurring ligands display micromolar affinity to PPARγ, an oxidized alkyl phospholipid hexadecylazelaoyl phosphatidylcholine (azPC) was shown to bind and activate the receptor at nanomolar range (Davies et al. [18]). This ligand is by far the most potent natural PPARγ ligand and has a similar affinity as synthetic ligand rosiglitazone. Unlike PPARγ, PPARα has been found to be activated by both saturated and unsaturated fatty acids, such as oleic acid, palmitic acid, linoleic acid, and arachidonic acid with micromolar affinities (Gottlicher et al. [19]). Although the lipoxygenase (LOX) metabolite 8(S)-HETE has been identified as a submicromolar ligand for PPARα, its low concentration in cells prohibits to classify it as a true natural ligand (Yu et al. [20]). It is more plausible that PPARα responds to the changes of whole-body free fatty acid pool (Chakravarthy et al. [21]) instead of high-affinity endogenous ligands. With a ligand selectivity intermediated between PPARγ and PPARα, the natural ligands of PPARβ include a list of saturated and unsaturated fatty acids, such as dihomo-γ-linolenic acid, EPA, and arachidonic acid. A number of eicosanoids (PGA1 and PGD2) were also identified to act on PPARβ (Forman et al. [22]). All of these natural occurring ligands bind PPARβ with micromolar affinities. Unlike natural ligands, the synthetic ligands of PPARs always have higher affinity with their receptors and have been widely used in clinical trials (Table 1). The most notable synthetic ligands of PPARγ are thiazolidinediones (TZDs) family which includes rosiglitazone, troglitazone, ciglitazone, pioglitazone, and englitazone (Willson et al. [23]). Among them, rosiglitazone was found to bind PPARγ with a high affinity (Kd ≈40 nM), while others are less potent ligands. TZDs have antidiabetic and insulin-sensitizing activities and have been widely used as prescription medicines. The fibrates, such as fenofibrate, clofibrate, and bezafibrate, are PPARα ligands and have been used in the treatment of hypertriglyceridemia. Through modification of atherogenic dyslipidemia, fibrates have also been shown to reduce coronary heart disease risk. Some PPARβ selective ligands have also been identified, such as L-165041 and GW 501516, and these compounds have certain serum lipid adjustment ability (Oliver Jr. et al. [24]).

The study of cannabinoids effects on PPARs started from the investigation of N-acyl ethanolamine OEA, a naturally occurring lipid derivative structurally related to anandamide which shares the anorectic property of other cannabinoids. Although OEA is a (albeit relatively low-affinity) CB receptor agonist and could enhance the activity of other endocannabinoids via an “entourage” effect by inhibiting the metabolism of other endocannabinoids, its regulation of feeding behaviour in rats appears to be due to its activation on PPARα (Fu et al. [25]; Fu et al. [26]). In vivo, OEA regulates feeding and body weight via a PPARα-dependent mechanism. OEA reduces body weight gain and triacylglycerol content in the liver and adipose tissues in subchronic treatments of diet-induced obese mice, but not in PPARα-knockout mice. Similarly, OEA induces lipolysis in both rats and wild-type mice but not in PPARα-knockout mice (Guzmàn et al. [27]).

Other cannabinoids, in addition to OEA, may serve as PPAR ligands as well. Recently, THC was found to activate one member of the PPAR family, PPARγ, in a concentration-dependent manner in transactivation assays in human embryonic kidney (HEK-293) cells (O'Sullivan et al. [28]). It also stimulated adipocyte differentiation in 3T3L1 cells, a well-accepted property of PPARγ ligands. It has also been demonstrated that THC can cause vasorelaxation through activation of PPARγ. Ajulemic acid (AJA) is a synthetic analog of THC which has no psychotropic activity in human or animal models but remains its analgesic and anti-inflammatory activities (Zurier et al. [29]). AJA has been approved recently for a phase II clinical trial for reduction of pain in humans. However, the biological effects of AJA cannot be altered by CB_1_ antagonist SR141716A and it does not show any binding ability to CB_1_/CB_2_ (Pertwee [6]). Recently, PPARγ was suggested as a possible candidate target site of AJA (Liu et al. [30]). The data reported demonstrate that AJA binds selectively to PPARγ in vitro and activate the transcriptional activity of PPARγ in cells. In addition, AJA induces 3T3L1 cells differentiation into adipocytes and inhibits IL-8 promoter activity in a PPARγ-dependent manner (Liu et al. [30]).

Polyunsaturated fatty acid amide anandamide was initially found to act as agonist of CB receptors. However, there are many other pathways involved in anandamide signalling. Anandamide activations on vanilloid receptor (Smart and Jerman [13]), T-type Ca^2+^ and K^+^ Task-1 ion channels have been demonstrated (Maingret et al. [31]). Catalyzed by a Ca^2+^-dependent phospholipase D, anandamide is produced through hydrolysis of the phospholipid precursor N-arachydonoyl-phosphatidylethanolamide (Di Marzo et al. [32]). Over the past ten years, there has been tremendous amount of effort put forth in attempts to characterize the mechanisms of anandamide transport (Fowler et al. [33]). After uptake into cells via diffusion or membrane transporters, anandamide can be hydrolysed by FAAH and produce arachidonic acid and ethanolamine (Di Marzo et al. [34]). Additionally, anandamide can be oxidized by various lipoxygenases (LOX) and cyclooxygenase-2 (COX-2), resulting in generation of ethanolamide analogs of hydroxyeicosatetraenoic acid (HETEs) and prostaglandins (prostamides) (Burstein et al. [35]). Through both hydrolysis and oxidation, anandamide brings some metabolites which may be potential PPARs ligands. Recently, anandamide also has been found to directly activate PPARγ (Bouaboula et al. [36]) and PPARα (Sun et al. [37]). Anandamide can bind PPARγ ligand binding domain directly and induce transcriptional activation of PPARγ in different cell types. Anandamide can stimulate 3T3L1 adipocyte differentiation and induce the expression of adipocyte differentiation markers such as C-EBPα and aP2 as well as PerilipinA, Acrp30, lipoprotein lipase, and PPARγ (Bouaboula et al. [36]). Anandamide has also been shown to directly bind to the ligand binding domain of PPARα by a CPA (cis-parinaric acid)-based fluorescence ligand binding assay and activate PPARα transcriptional potency in the HeLa cell line (Sun et al. [37]). It is worthy to mention that in the same systems, most endocannabinoids (OEA, anandamide, noladin ether, and virodhamine) show similar binding and transcription activity on PPARα with one important exception PEA, a saturated analogue of OEA and anandamide. PEA was found to activate PPARα in cultured cells and to induce the expression of PPARα. In vivo, PEA attenuates inflammation in wild-type mice but not in PPARα-knockout mice (Lo et al. [38]). However, PEA cannot displace CPA from PPARα ligand binding domain which suggests an indirect mechanism (Sun et al. [37]). In addition to anandamide, 2-AG and noladin ether, the nonhydrolyzable analog of 2-AG, have been demonstrated to mediates the suppression of IL-2 through PPARγ (Rockwell et al. [39]). The inhibition of IL-2 expression by 2-AG and noladin ether is independently of CB_1_/CB_2_, since similar suppression of IL-2 by 2-AG and noladin ether was observed in splenocytes derived from CB_1_/CB_2_-null mice and CB_1_/CB_2_ antagonists failed to block inhibition of IL-2 by 2-AG. 2-AG and noladin ether increased PPARγ transcriptional potency in 3T3L1 cells, forced 3T3L1 adipocyte differentiation, and induced the expression of adipocyte differentiation marker aP2. The involvement of PPARγ was further confirmed by the fact that PPARγ-specific antagonist 2-chloro-5-nitro-N-(4-pyridyl)-benzamide (T0070907) blocked 2-AG and noladin ether-mediated IL-2 suppression. It is worthy to note that NS398, a COX-2 specific inhibitor, blocked 2-AG and noladin ether-mediated IL-2 suppression as well, suggesting the requirement for COX-2 metabolism for the inhibition of IL-2 (Rockwell et al. [39]). Cannabinoids effects on the PPARβ subtype have not been comprehensively studied yet, probably due to shortage of apparent pharmacological significance.

**Figure 2:**
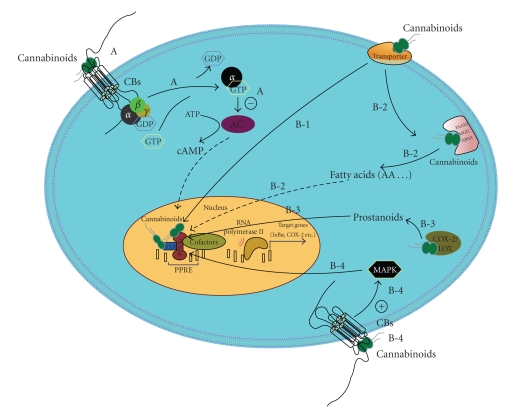
A. Classical cannabinoids effects on CB receptors. B. Possible pathways involved in cannabinoids effects on PPARs, B-1. Some cannabinoids act as genuine ligands of PPARs or RXRs, B-2. Enzymes involved in hydrolysing endocannabinoids may generate ligands of PPARs, B-3. Endocannabinoids can be metabolised by COX-2 and LOX and generate ligands of PPARs; B-4. CB receptors activation leads to the stimulation of MAPK pathway which may be reflected by PPARs (AA: arachidonic acid; AC: adenylate cyclase).

Cannabinoids effects on PPARs involve a series of enzymes, proteins, and several interlaced pathways (Figure 2). In order to directly act on nuclear transcriptional factors PPARs, exogenous cannabinoids need to pass through plasma membrane and be transported into nucleus which may involve certain membrane and intracellular transporters. Genuine ligands of PPARs bind and change the structure of the nuclear transcriptors and form asymmetrical dimers with RXRs. This binding changes the conformation of the PPARs and induces binding to the PPRE, which have been found in numerous PPAR-inducible genes. With the help of certain coactivators or corepressors, genes transcription is induced or suppressed. However, we still cannot rule out that cannabinoids effects could be indirect through the binding of other cellular targets which in turn induces PPARs activation. For example, FAAH, monoglyceride lipase (MGL), and N-palmitoylethanolamine-selective acid amidase (NPAA) are three key enzymes involved in hydrolysing endocannabinoids (Vandevoorde and Lambert [40]). Degradation of cannabinoids by these enzymes may generate novel ligands of PPARs. When present in large quantities, substrates of these enzymes can also compete with endocannabinoids which are bona fide ligands of PPARs from degeneration. PEA has no binding ability with PPARγ and PPARα in vitro, but it displays anti-inflammatory property in a PPARα-dependent pathway in a mouse model (Lo et al. [38]). NPAA is the PEA selective hydrolase which presumably contributes to PEA activation on PPARα via “entourage” effects (Ueda et al. [41]). Endocannabinoids can also be metabolised by COX-2 and LOX and produce prostanoids which are proved PPARs activators (Burstein et al. [35]). Another possible PPAR-related target of cannabinoids is RXR. It has been shown that polyunsaturated fatty acids including arachidonic acid bind and activate RXRα (Lengqvist et al. [42]). Thus, endocannabinoids, especially anandamide may also activate RXRs that heterodimerize with PPARs. Even cannabinoids effects on CB_1_/CB_2_ can potentially lead to the activation of PPARs. Ligand-binding to CB_1_/CB_2_ receptors elicit a concentration-dependent increase in the activity of mitogen-activated protein kinase (MAPK), which is independent of adenylate cyclase inhibition (Rueda et al. [43]). PPARs activation can be regulated through direct phosphorylation by different members of MAPK family or by reflecting MAPK modification of other cellular components that interfere with PPARs (Gelman et al. [44]). Considering the complexity of the mechanisms, cannabinoids effects on PPARs need to be investigated attentively in certain circumstances.

The cannabinoids influences on PPARs consist of considerable theoretical and therapeutic significances. Although many effects of cannabinoids can be explained though their action on membrane-associated G protein-coupled receptors CB_1_/CB_2_ and related downstream signalling cascades; nuclear receptors PPARs provide an additional mechanism for cannabinoids regulation of gene transcription which may associate with their long-term exposure consequences. Many therapeutic effects of cannabinoids including management of glaucoma, rebel of inflammatory and neuropathic pain, amelioration of certain types of cancer, and various kinds of motor dysfunction associated with multiple sclerosis, spinal cord injury, and ischemic stroke can be connected with PPARs as well (Pertwee [45]). In summary, there is strong evidence to suggest that some cannabinoids can act on PPARs through either direct or indirect pathways. These discoveries not only broaden the promising usage of cannabinoids as therapeutic agents, but also support PPARs as new targets for some neuroprotective treatment.

## Figures and Tables

**Table 1:** Nature and synthetic ligands of PPARs.

PPARγ	PPARα	PPARβ

Nature ligands		

Linoleic acid	Palmitic acid	Fatty acids
Arachidonic acid	Linoleic acid	Dihomo-γ-linolenic acid
15d-PGJ2	Stearic acid	EPA
9-HODE	Palmitoleic acid	Arachidonic acid
13-HODE	Oleic acid	Eicosanoids
15-HETE	Linoleic acid	—
—	Arachidonic acid	—
—	Eicosapentaenoic	—
—	8(S)-HETE	—

Synthetic ligands		

TZDs	WY-14643	L-165041
JTT-501 (isoxazolidinedione)	Clofibrate	GW-501516
GW-7845	Gemfibrozil	NSAIDs (antagonist)
CDDO	Nafenopin	—
BADGE (antagonist)	Bezafibrate	—
LG-100641 (antagonist)	Fenofibrate	—
